# Proteomic Analysis Shows Constitutive Secretion of MIF and p53-associated Activity of COX-2^−/−^ Lung Fibroblasts

**DOI:** 10.1016/j.gpb.2017.03.005

**Published:** 2017-12-13

**Authors:** Mandar Dave, Abul B.M.M.K. Islam, Roderick V. Jensen, Agueda Rostagno, Jorge Ghiso, Ashok R. Amin

**Affiliations:** 1 Department of Rheumatology, New York University Hospital for Joint Diseases, New York, NY 10003, USA; 2 Department of Science, STEM Division, Union County College, Cranford, NJ 07016, USA; 3 Department of Genetic Engineering and Biotechnology, University of Dhaka, Dhaka 1000, Bangladesh; 4 Department of Biological Sciences, College of Science, Virginia Tech, Blacksburg, VA 24060, USA; 5 Departments of Pathology, New York University School of Medicine, New York, NY 10003, USA; 6 Department of Bio-Medical Engineering, Virginia Tech, Blacksburg, VA 24060, USA; 7 RheuMatric Inc., Blacksburg, VA 24061, USA

**Keywords:** MIF, p53, Cyclooxygenases, Cancer, Proteomics

## Abstract

The differential expression of two closelyassociated **cyclooxygenase** isozymes, COX-1 and COX-2, exhibited functions beyond eicosanoid metabolism. We hypothesized that *COX-1* or *COX-2* knockout lung fibroblasts may display altered protein profiles which may allow us to further differentiate the functional roles of these isozymes at the molecular level. Proteomic analysis shows constitutive production of macrophage migration inhibitory factor (**MIF**) in lung fibroblasts derived from COX-2^−/−^ but not wild-type (WT) or COX-1^−/−^ mice. MIF was spontaneously released in high levels into the extracellular milieu of COX2^−/−^ fibroblasts seemingly from the preformed intracellular stores, with no change in the basal gene expression of *MIF*. The secretion and regulation of MIF in COX-2^−/−^ was “prostaglandin-independent.” GO analysis showed that concurrent with upregulation of MIF, there is a significant surge in expression of genes related to fibroblast growth, FK506 binding proteins, and isomerase activity in COX-2^−/−^ cells. Furthermore, COX-2^−/−^ fibroblasts also exhibit a significant increase in transcriptional activity of various regulators, antagonists, and co-modulators of **p53**, as well as in the expression of oncogenes and related transcripts. Integrative Oncogenomics Cancer Browser (IntroGen) analysis shows downregulation of COX-2 and amplification of MIF and/or p53 activity during development of glioblastomas, ependymoma, and colon adenomas. These data indicate the functional role of the MIF-COX-p53 axis in inflammation and **cancer** at the genomic and **proteomic** levels in COX-2-ablated cells. This systematic analysis not only shows the proinflammatory state but also unveils a molecular signature of a pro-oncogenic state of COX-1 in COX-2 ablated cells.

## Introduction

The tumor suppressor gene *TP53* averts cancer by regulating several cellular functions. These include growth arrest, apoptosis, senescence, and oncogene activation [1], [2]. Mutation(s) in *TP53* and/or loss of wild-type *TP53* can result in a gain of transforming and neoplastic activity in cells [1], [2]. The p53 tumor suppressor protein functions closely with its negative regulator E3 ubiquitin ligase or mouse double minute 2 homolog (MDM2), which limits its tumor suppressor functions in normal unstressed cells [1], [2]. Cellular stress, such as DNA damage, blocks the binding of MDM2 to p53, resulting in increased levels of p53 that promote cell cycle arrest to repair damaged DNA or apoptosis of the cell to avoid transfer of damaged DNA to daughter cells [1], [2]. Also, p53 protein interacts with numerous other proteins, resulting in a broad range of physiologic and oncogenic processes [1], [2].

Eicosanoids including prostaglandins (PGs), leukotrienes (LTs), and thromboxanes (TXs) are essential mediators of inflammation, inflammation resolution, pain, and fever [3], [4]. PGs, which exhibit diverse functions [3], [4], [5], [6], can be synthesized by the constitutive cyclooxygenase-1 (COX-1) and/or the inducible isoform COX-2 [3], [4]. PGs and TXs are together referred to as prostanoids, which can be inhibited by non-steroidal anti-inflammatory drugs (NSAIDs). Previous work from our lab has shown that COX-1 or COX-2-ablated fibroblasts exhibit differential synthesis of prostanoids, together with alterations in gene expression and cellular functions [4].

COXs and p53 share common regulatory mediators and have complex relationships [7]. They are both sensitive to redox changes [2], [7], nitric oxide [2], [7], hypoxia [2], [7], [8], [9], [10] and oncogene activation [1], [2], [7], [8], [9], [10]. Moreover, they together participate in RNA transcription [2], [7], DNA synthesis and replication, [1], [2], [7] as well as inflammation [2], [3], [6], [7]. The differential expression of p53 and COX-2 is evident in many neoplastic conditions and cancers [1], [2], [7], [8], [9]. For instance, expression of p53 (but not mutant p53) can suppress the expression of *COX-2* (by 85%) via the p53-TATA-binding protein (TBP) in murine embryo fibroblast-derived cell lines [11]. Nevertheless, COX-2 can inactivate p53 via protein–protein interactions [12]. COX-2 also exhibits PG-independent functions in fibroblasts [4], prostate cancer cells [13], breast cancer cells [14], and squamous carcinomas [15]. Thus, COX-2 and p53 exhibit a mutual interaction depending on the cell type. Indeed, NSAIDs and Coxibs have been reported to provoke growth arrest and apoptosis in a COX-2-independent fashion by increasing the levels of p53 [7], [9], [10].

Macrophage migration inhibitory factor (MIF) exhibits cytokine-like activities [16], [17], [18], [19], and it signals through CD74 and CD44 receptors, resulting in the secretion of IL-1, IL-6, IL-8, TNF-α, matrix metalloproteinases, and COX-2-related products [17], [18], [19], [20]. MIF is abundantly expressed and stored in the cytoplasm [16], [20]. A non-classical protein secretion pathway allows the release of preformed MIF from cytoplasmic pools without alterations in the mRNA expression levels of *MIF* [20], [21]. MIF is reported to be upregulated in virtually all stages of neoplasia in most types of cancers and metastatic conditions [16], [18], [21], [22], [23], [24]. Moreover, upregulated expression of MIF and COX-2 is reported in several tumors, most notably in small-cell lung carcinoma and colon cancer [7], [9], [16], [18]. Co-expression of COXs and MIF is also implicated in various chronic inflammatory conditions such as sepsis, asthma, arthritis, dermatitis, atherosclerosis inflammatory, cell-mediated immunity, and innate immunity, as well as different functions of macrophages, such as tumorigenic activity, chemotaxis, and phagocytosis [6], [16], [17], [18], [19], [20], [21], [22], [23], [24], [25]. Anti-MIF treatment efficiently suppresses tumor-associated angiogenesis, tumor growth, and autoimmune diseases such as human rheumatoid arthritis [16], [21] and cancer [26]. On the other hand, joint inflammation is significantly decreased in *MIF*-knockout mice as compared to normal mice [16], [27]. These studies demonstrate the multiple functional properties of MIF as a cytokine and hormone [16], [17], [18], [19], [20], [21]. The N-terminus of MIF may also function as a phenylpyruvate tautomerase which catalyzes 2-carboxy-2,3-dihydroindole-5,6-quinone (dopachrome) into 5,6-dihydroxyindole-2-carboxylic acid (DHICA) [22]. This study describes the proteomic analysis of WT, COX-1^−/−^, and COX-2^−/−^ cells, which unravels an unanticipated PG-independent increase and release of MIF in COX-2^−/−^ cells. Surprisingly, the spontaneous increase in eicosanoid metabolism by the homeostatic COX-1 activity in COX-2^−/−^ cells is also allied with a rise in expression of oncogenes, p53 activity and related transcripts normally induced during cell-stress and cancer. Collectively, this study for the first time shows eradication of *COX-2* activity generates a pro-inflammatory and pro-oncogenic state at the molecular level in COX-2^−/−^ cells.

## Results

**Figure 1:**
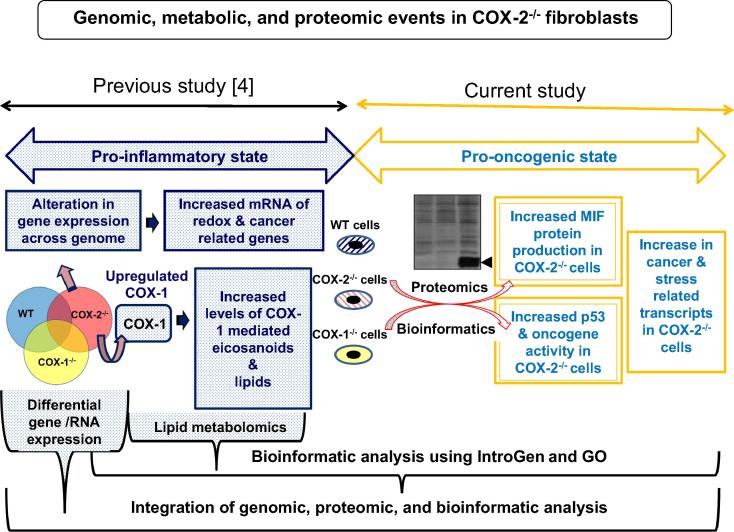
**Flow-chart of a systems approach to translational genomics of COX-2^−/−^ cells** COX-2^−/−^ cells have increased expression of *COX-1*, as well as genes related to redox, cancer, and several other mRNAs within and outside the eicosanoid metabolism, together with an increase in COX-1 mediated eicosanoids [4]. Proteomic and bioinformatic analysis showed an increase in MIF secretion and p53 activity. The combined study identified the COX/p53/MIF axis in COX-2^−/−^ cells, resulting in pro-inflammatory and pro-oncogenic states in lung fibroblasts at the molecular level. COX, cyclooxygenase; MIF, macrophage migration inhibitory factor.

Chronic inflammation can lead to numerous types of cancer [1], [2], [7], [8]. We combined various approaches and tools of translational genomics trying to decipher the complicated relationship of COXs in both inflammation and oncogenesis at the cellular level in fibroblasts. Our previous work indicated changes in gene expression, metabolomics of eicosanoids, and redox reactions in COX-1^−/−^ and COX-2^−/−^ fibroblasts [4]. The increased in COX-1 mediated inflammatory eicosanoids in COX-2^−/−^ cells (similar to IL-1β activated WT cells) did not adequately explain the alteration of global gene expression within and outside eicosanoid metabolism especially gene expression related to oncogenic activity [4]. This prompted us to examine the changes in protein expression in COX-1^−/−^ and COX-2^−/−^ cells. Figure 1 summarizes the strategy utilized in the previous [4] and current studies in these fibroblasts.

### Proteomic analysis of WT, COX-1^−/−^, and COX-2^−/−^ mouse fibroblast cell lines

**Figure 2:**
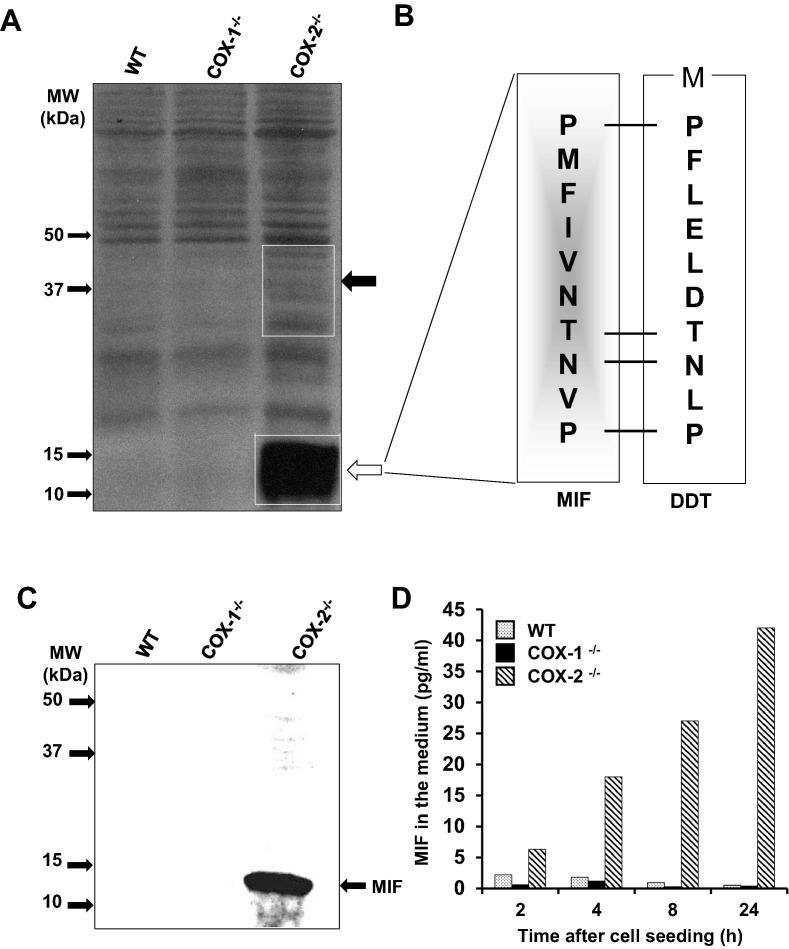
**MIF overexpression in COX-2^−/−^ lung fibroblast cultures** **A.** Proteomic identification. 20 µg of the total cytosolic cell lysates from WT, COX-1^−/−^, and COX-2^−/−^ cells were separated on a 12% SDS–PAGE gel and visualized by silver staining. The white boxes indicate the differentiallyexpressed proteins. **B.** Protein sequencing. The overexpressed 10−15 kDa protein band from COX-2^−/−^ cells was subjected to N-terminal sequencing and identified as a macrophage migration inhibitory factor (MIF). The N-terminal sequence of the homologous (DDT) protein is presented for comparison [22]. The data represent one of the two similar experiments. **C.** Western blot analysis. 30 μg of total cytosolic cell extracts from WT, COX-1^−/−^, and COX-2^−/−^ cells were separated on 12% SDS–PAGE gel and subjected to immunoblotting using an anti-MIF antibody. The arrow shows the band recognized by the anti-MIF antibody. The data represent one of the three similar experiments. **D.** Quantitation of MIF in cell supernatant. Equal amounts of cell supernatants from WT, COX-1^−/−^, and COX-2^−/−^ cells was taken at various time points 0–24 h after seeding. The amount of MIF was estimated using ELISA. The data represent one of the two similar experiments. DDT, D-dopachrome tautomerase.

We compared the protein expression in lung fibroblast cell lines derived from wild-type (WT) C57BL/6J mice and COX-1^−/−^ and COX-2^−/−^ mice. SDS–PAGE gel analysis and silver staining showed that the cytosolic fraction from all three cell lines had a similar profile of protein bands (Figure 2A). However, several protein bands with a molecular weight below 50 kDa were quantitatively and/or qualitatively distinct in COX-2^−/−^ lysates as compared to WT and COX-1^−/−^ cells (indicated by boxes and arrows, Figure 2A). Among these, the most prominent difference was the protein band(s) of 10–15 kDa, which were detected to be greatly overexpressed in the COX-2^−/−^ cell lysates.

To further identify these 10–15 kDa proteins, the cell lysates procured from the COX-2^−/−^ cells were separated and electrotransferred to a PVDF membrane. The resulting protein region corresponding to the 10–15 kDa was subjected to N-terminal sequencing. As a result, a single sequence: PMFIVNTNVP was consistently retrieved from two different batches of COX-2^−/−^ cell lysates, indicating that if other components with the same electrophoretic mobility co-existed, their concentration had to be below the sensitivity of the Edman degradation procedure. The PMFIVNTNVP showed 100% identity with the first 10 N-terminal amino acid residues of mouse MIF (GenBank accession No. P34884) and was not related to the highlyhomologous protein, D-dopachrome tautomerase (DDT) [22], also a member of the MIF family [22] (Figure 2B). Immunoblotting analysis using anti-MIF antibodies (Figure 2C) revealed a robust MIF signal in the 10–15 kDa region of COX-2^−/−^ cell lysate but not in COX-1^−/−^ or WT cell lysate. These results further confirmed the protein identity as MIF.

The secretory MIF protein exhibits both autocrine and paracrine functions via CD74, CXCR2, and CXCR4 receptors [18], [19], [20]. Therefore, we analyzed the release of MIF in the supernatants of wild-type and COX-1^−/−^, COX-2^−/−^ cells. Given that the extracellular MIF may be diluted in the medium compared to cell lysates, we used a more sensitive method of sandwich ELISA, which could directly and specifically measure low levels [pg/ml] of MIF, to detect the secreted MIF in the medium. As shown in Figure 2D, there were low levels (∼0–2 pg/ml) of MIF in WT and COX-1^−/−^ cell supernatants. In contrast, we detected the spontaneous and continuous release of MIF (∼40 pg/ml) in the medium of COX-2^−/−^ cells, which was about 20-fold as high as that detected in COX-1^−/−^ and WT cells. These experiments showed increased production and secretion of MIF in the absence of functional COX-2 (Figure 2).

### Distinct functional relationship of MIF in *COX-1*- and *COX-2*-ablated cells

To explore the functional correlation between MIF and *COX* expression, we collected the publicly available information (from KEGG database) on metabolic pathways and molecular functions associated with MIF, actively. We used the gene expression data procured from COX-1^−/−^, COX-2^−/−^, and IL-1β-stimulated WT fibroblast cells [4] for GO-enrichment analysis as described in Material and Methods. As shown in Figure S1A, MIF and IL-1β stimulated WT cells shared a general role in biological processes involving inflammation. COX-1 and COX-2 expression is linked to distinct biological processes in conjunction with MIF as shown in Figure S1A. The GO molecular function analysis demonstrated common chemoattractant activity among COX-1^−/−^, COX-2^−/−^, WT + IL-1β, and MIF (Figure S1B). However, an increase in the isomerases and FK506 binding protein activity was only revealed with the upregulation of MIF in COX-2^−/−^ cells (Figure S1C) (http://en.wikipedia.org/wiki/FKBP). These data highlight the differential and functional connection between MIF and COX-1 in COX-2^−/−^ cells.

### MIF secretion is independent of upregulated *MIF* gene expression and levels of PGE_2_

We have previously identified over 532 transcripts with increased expression (FC > 1.75) in COX-2^−/−^ fibroblasts [4]. We thus tested factors that may regulate the expression and secretion of MIF. The relative expression of *MIF* was similar to that of the housekeeping genes in all cell groups examined in this study (Figure S2). However, expression of *Gstt1* and *Ddt* that are transcribed from the *MIF*-*Ddt*-*Gsst* cluster located on chromosome 10 (Figure S3) was upregulated in COX-2^−/−^ cells as compared to WT cells (Figure S2). These preliminary observations do not support a direct contribution of increased *MIF* mRNA levels to the upregulated production of MIF in COX-2^−/−^ cells.

**Figure 3:**
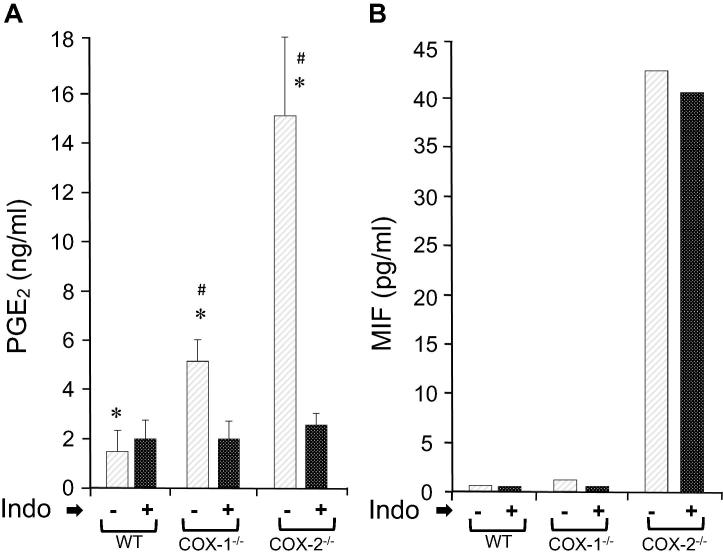
**Production of PGE_2_ and MIF in the presence of indomethacin** **A.** Equal amounts of WT, COX-1^−/−^, and COX-2^−/−^ cells were grown for 24 h in the absence or presence of 5 µM of Indo, and the cell supernatants were used to estimate the amount of PGE_2_ and MIF released into the medium using RIA and ELISA, respectively. Data are expressed as mean ± SD (*n* = 3). The Wilcoxon–Mann–Whitney test was used for statistical analysis. ^*^Indicates significant difference in the levels of spontaneously released PGE_2_ between WT and COX-1 or COX-2 ablated cells (*P* < 0.05), whereas ^#^ indicates significant difference in the levels of spontaneously released PGE_2_ between COX-1^−/−^ and COX-2^−/−^ cells (*P* < 0.05). No significant difference was detected between WT, COX-1^−/−^, and COX-2^−/−^ cells treated with Indo. **B.** The MIF levels in the absence and presence of indomethacin in WT, COX-1^−/−^, and COX-2^−/−^ cells were estimated using a MIF-specific ELISA. Indo, indomethacin; PGE_2_, prostaglandin E_2_.

PGE_2_ accounts for ≥ 80% of the PGs synthesized by COX-1 or COX-2 cyclooxygenases [3], [4], [28]. Increased levels of PGs inhibit the expression of MMP-1 but promote MMP-13 and cytokine secretion, whereas a decrease in EP2 receptor expression reduces collagen synthesis in fibroblasts [5], [29]. We therefore examined whether changed levels of PGs in COX-2^−/−^ cell contribute to the increased secretion of MIF. Administration of COX-1 inhibitor SC560 and COX-2 inhibitor Celebrex (at IC_50_ concentrations) significantly inhibited the production of PGE_2_ via COX-1 in COX-2^−/−^ cells and via COX-2 in COX-1^−/−^ cells, respectively (data not shown). We then used a non-selective COX inhibitor indomethacin to inhibit synthesis of all PGs by COX-1 and COX-2 [4]. As shown in Figure 3A, indomethacin treatment led to a decreased accumulation of PGE_2_ in both COX-1^−/−^ and COX-2^−/−^ cells, with the levels similar to basal levels observed in the WT cells. Similarly, we examined the MIF protein expression in these cells and found low basal expression (0.6 pg/ml) in WT and COX-1^−/−^ (1.3 pg/ml) cells. However, the levels of MIF in COX-2^−/−^ cells remained as high as ≥ 40 pg/ml, in the absence and presence of indomethacin (Figure 3B). These data indicate that reduced synthesis of PGs in COX-2^−/−^ cells had no significant impact on the secreted MIF in the medium.

**Figure 4:**
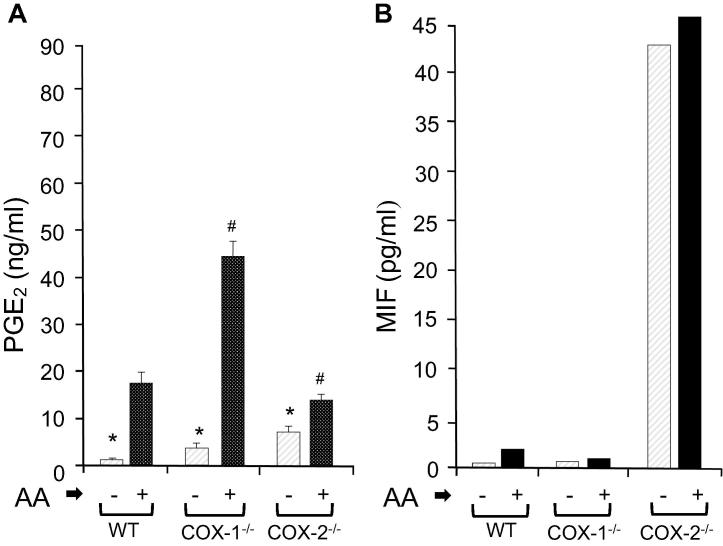
**Production of PGE_2_ and MIF in the presence of arachidonic acid** **A.** Equal amounts of WT, COX-1^−/−^, and COX-2^−/−^ cells were grown for 24 h in the absence or presence of 0.5 µM of AA, and the cell supernatants were used to estimate the amount of PGE_2_ and MIF. Data are expressed as mean ± SD (*n* = 3). The Wilcoxon–Mann–Whitney test was used for statistical analysis. ^*^Indicates significant difference in the levels of spontaneously released PGE_2_ between WT and COX-1 or COX-2 ablated cells (*P* < 0.05), whereas *^#^* indicates significant difference in PGE_2_ between COX-1^−/−^ and COX-2 in the presence of AA (*P* < 0.05). **B.** The MIF levels in the absence and presence of AA in WT, COX-1^−/−^, and COX-2^−/−^ cells were estimated using a MIF-specific ELISA. AA, arachidonic acid.

Increased levels of PGE_2_ are known to stimulate NFκB and cAMP-mediated signaling, gene expression, DNA methylation, as well as the production of IL-6 and IL-8 [3], [4], [5], [9]. We thus tested the effects of elevated levels of PGE_2_ on MIF secretion. Arachidonic acid (AA) is a rate-limiting step in the biosynthesis of leukotrienes (LTs) and PGs [3], [4], [5], [9], and its availability can serve as a means to augment the endogenous PG synthesis above basal levels. As expected, the addition of 0.5 µM AA significantly augmented the production of PGE_2_ above the basal levels (*P* < 0.05) in all three cell groups (Figure 4A). However, there were no changes in the levels of MIF in COX-1^−/−^ or COX-2^−/−^ cell supernatants in the presence or absence of AA (Figure 4B). An increase or a decrease in PGE_2_ levels did not affect the accumulation of MIF in COX-2^−/−^ cells. These observations suggest the constitutive production of MIF in COX-2^−/−^ cells was dependent on the absence of *COX-2* and upregulation of *COX-1* gene but independent of the catalytic activity of COX-mediated PGE_2._

### Upregulation of oncogenes and related transcripts in COX-2^−/−^ cells

**Figure 5:**
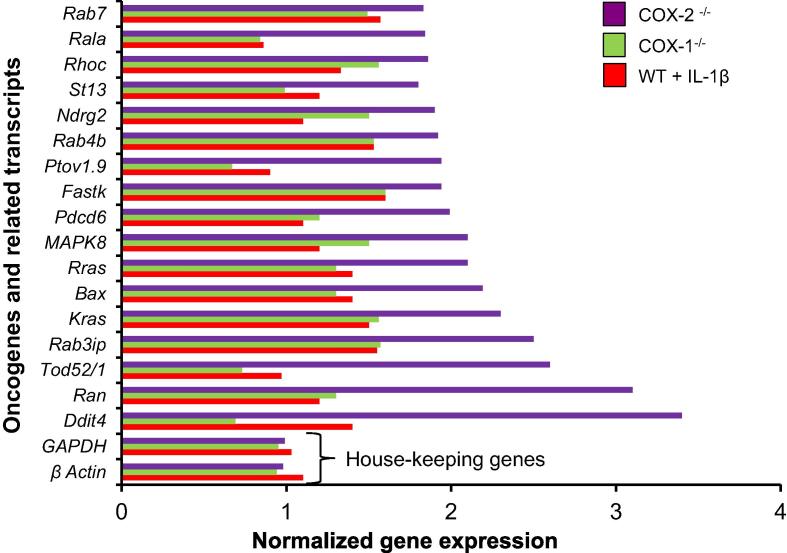
**Gene expression array of oncogenes in IL-1β-treated WT, COX-1^−/−^, and COX-2^−/−^ cells**Gene expression arrays of (WT), IL-1β stimulated WT (WT + IL-1β), COX-1^−/−^, and COX-2^−/−^ cells were executed as previously reported [10]. The graph shows the differential expression of oncogenes and related transcripts. The mRNA expression levels were plotted in arbitrary units as a ratio of WT, after normalizing with the expression levels of housekeeping genes *actin* and *GAPDH*. The full forms of the abbreviated gene names are available on http://www.genecards.org/

Our preliminary observations suggested that the COX-2 null cells may exhibit oncogenic activity [4]. This observation was propped by a sixfold surge in the expression of the gene encoding MDM2, a principal antagonist of p53 [1], [2] in COX-2 null cells. We then employed three different approaches to confirm our preliminary observations. First, we utilized our gene expression data [4] to identify oncogenes and the related transcripts that are highly expressed in COX-2^−/−^ fibroblasts. Figure 5 shows the expression of 17 oncogenes and related transcripts that were upregulated in COX-2^−/−^ to a greater extent than that in COX-1^−/−^ and IL-1β-stimulated WT cells. These observations show a pattern of common functional genes that are not only co-modulated with the expression of p53 but are also known to be upregulated in transformed cells of lung, colon, and prostate cancers [1], [2], [10], as well as COX-2 ^−/−^ fibroblasts as shown in this study.

### Regulation of *PTGS1*, *PTGS2*, and *TP53* in cancers

To further understand the “pro-oncogenic state” of COX-2 nulls cells, we next examined the gene expression of *COX-1* and *TP53* in various tumors using bioinformatics analysis. We performed the analysis utilizing data from the Integrative Oncogenomics (IntOGen) system for the COX-1 (*PTGS1*), COX-2 (*PTGS2*), and p53 (*TP53*) genes. As shown in Figure S4, there was a significant change in the expression of *PTGS1* in diverse tumors such as the brain, kidney, nasopharyngeal, colon, leukemia, and other hematopoietic reticuloendothelial systems and various related pathophysiological conditions. Among these, changes in *PTGS1* and *TP53* were commonly observed in glioblastomas, nasopharyngeal, and colon cancer. Moreover, *PTGS1*, *PTGS2*, and *TP53* activity was all significantly increased in colon adenoma, for which the PG-independent effects have been recognized for almost two decades [6], [9], [13], [14], [15].

### Modulation of p53 targets in COX-2^−/−^ cells

**Figure 6:**
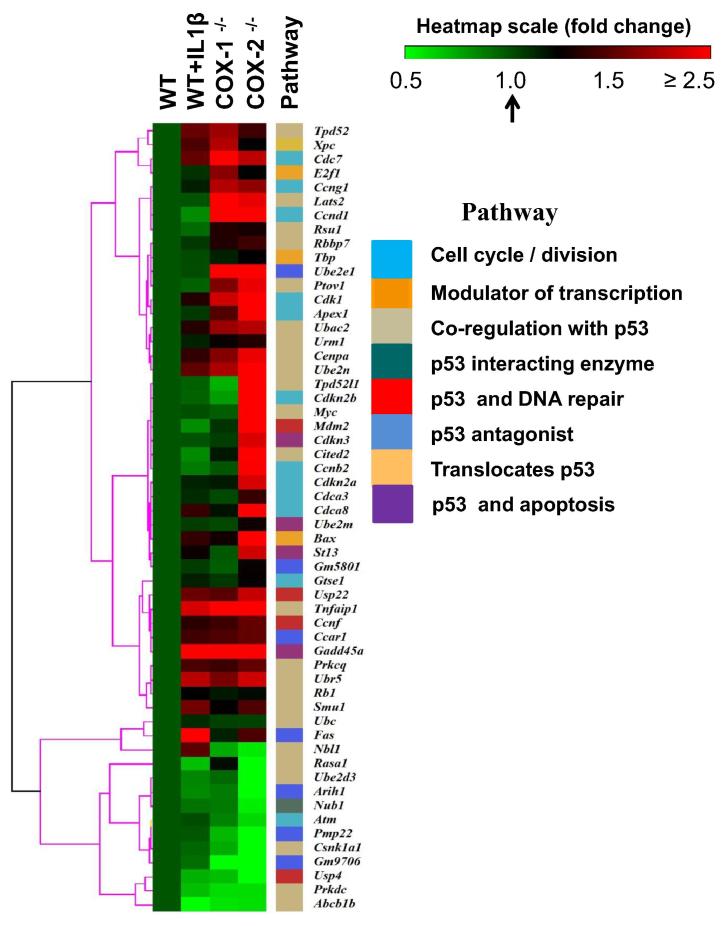
**Gene expression of p53-related genes in COX-1 and COX-2 ablated cells** The differential gene expression of p53-associated transcripts termed as p53-targets is presented as a heat map for WT, WT + IL-1β, COX-1^−/−^, and COX-2^−/−^ cells. Fold change of expression is indicated in color gradient (color toward red indicates higher gene expression levels and color toward green indicates lower expression levels as compared to WT). The differentially expressed genes were grouped into distinct pathways, which are color-coded based on the direct or indirect interactions with p53. A detail list of these transcripts and the associated fold changes is shown in Table S1.

To characterize the pro-oncogenic state of COX-2^−/−^ cells, we lastly identified changes in transcripts (and targets) that influence p53 functions. As shown in Table S1, we detected 56 p53-influencing targets from our gene expression arrays [4]. These targets included p53-related pathways such as cell cycle/division, co-modulation of transcription, co-regulation of p53, modulation of p53-interacting proteins, DNA repair proteins, and factors involved in translocation of p53 as shown in Table S1. Table 1 shows enrichment analysis of p53 target genes in COX null cells, and IL-1β-treated cells. Compared to WT cells, expression of 5, 11, and 32 transcripts was significantly changed in IL-1β-treated WT, COX-1^−/−^, and COX-2^−/−^ cells, respectively. These observations imply an increased *p53*-related activity in COX-2^−/−^ cells. Expression pattern of these p53 targets is shown as a heat map in Figure 6 with the corresponding pathways and functions in all the matching cell groups. In summary, these bioinformatics analyses reveal overlapping observations and further strengthen the proposed pro-oncogenic state of COX-2^−/−^ fibroblasts.

**Table 1:** **Enrichment of p53 target genes in COX-1^−/−^, COX-2^−/−^, and IL-1β treated WT cells**

**Group**	**Average No. of expected random targets**	**No. of actually-observed deregulated targets**	**Corrected *P* value**
WT + IL-1β	1.22	5	0.0075
COX-1^−/−^	2.61	11	0.000048
COX-2^−/−^	4.67	32	1.70E−020

*Note*: 56 genes encoding proteins that are known to interact with p53 [1], [2], [9], [18] were prepared as a target module [4]. The module was tested to see whether the representative genes [4] were over-represented for functions in this module using Gitools with multiple tests corrected P values (FDR) for enrichment. A detail list of these 56 genes is presented in Table S1.

### Role of MIF and COX-1 in oncogenesis

The role of p53 and its associated isoforms and collaborators in cancer is irrefutable [1], [2], [9]. The inability to identify and develop a drug for intervention could be attributed to the functional complexity of p53 [1], [2]. The observed upregulation of a multifunctional MIF [16], [19] and the increased functional activity of p53 in this study introduces another double-edged sword [1], [2] in complex metabolic and genomic activity induced in COX-2^−/−^ fibroblasts [3], [7], [9]. The upregulation of sterile inflammation, MIF, as well as gene expression of glutathione *S*-transferase (GST) and oncogene-related transcripts in COX-2^−/−^ cells further supports a pro-inflammatory and -oncogenic state of COX-2^−/−^ fibroblasts as compared to WT cells. MIF is theta-class GST homologs [16], [22], [30]. GSTs are also biomarkers of cancer drug resistance [31].

We further applied IntOGen (http://www.intogen.org) to examine the phenotypic characteristics of tumors with significantly modulated COX-2 and MIF. Table S2 shows the upregulation or downregulation of COX-2 in numerous cancers. *COX-2* gene expression is significantly upregulated in cancers of the adrenal glands and papillary adenocarcinomas. The *COX-2* expression is ominously downregulated in numerous types of cancers of the adrenal glands, skin, stomach testis, brain (*e.g.*, glioblastoma) bladder, kidney (*e.g.*, nephroblastoma and renal cell carcinoma), and ovary (*e.g.*, cystadenocarcinoma).

The transcriptomic status of MIF alone was not reported in IntOGen with related microarray studies. We thus examined the phenotypic characteristic cancers with significant loss of COX-2 activity and significant gain of MIF activity (as observed in COX-2^−/−^ cells) from the IntOGen database. As shown in Table S3, there was a broad range of cancers with upregulated MIF. However, a significant increase in MIF with a significant concurrent loss of *COX-2* activity was only found in cancers of nervous systems and brain (*e.g.*, ependymoma and neuroblastoma).

Taken together, our experimental evidence of dysfunctional COX-2 and amplification of MIF overlaps with a significant and substantial signature for oncogenesis based on the analysis of IntOGen data, bringing together the action of the multifunctional MIF and p53 in COX-2^−/−^ cells.

## Discussion

Our holistic study presented a reliable gene expression analysis with stringent bioinformatics and internal controls [32] as previously reported [4]. MIF has exhibited a bewildering and tantalizing history of rediscoveries and controversies regarding its role in inflammation, innate immunity, and neoplastic activity [17], [18], [20]. The present study highlights MIF-COX-p53 axis with the constitutive secretion of MIF, which operates in the absence of a function *COX-2* and upregulated *COX-1* gene, but in a PG-independent manner.

### Constitutive production of MIF is released from intercellular store in COX-2^−/−^ fibroblasts

Immunoblotting analysis showed increased levels of intracellular MIF in COX-2^−/−^ cell lysates as compared to WT or COX-1^−/−^ cells, despite the similar levels of mRNA expression of *MIF* in these cells. The possibility of other proteins other than MIF present in the 10–15 kDa region of COX-2^−/−^ cells could not be ruled out with the methods used in this study. These issues can be resolved using more sensitive methods such as LC–MS/MS. MIF lacks N-terminal or the internal secretory signal sequence like IL-1 and basic FGF [18], [20]. Instead, the secretion of MIF via intracellular pools is facilitated by a specific non-classical pathway that involves the p115 protein in the Golgi apparatus [18], [20]. Previous studies have shown that arthritis-affected synovial fibroblasts, virus-infected cells, adipose tissues, and endotoxemia-affected macrophages have increased levels of MIF with no significant surge in *MIF* mRNA expression [18], [20], [33], [34], [35], [36]. These and our observations suggest that a substantial amount of MIF is available in intracellular stores in COX-2^−/−^ fibroblasts.

### MIF secretion is independent of PGE_2_ levels

MIF can increase PLA_2_ activity and eicosanoid synthesis via the protein-A dependent pathway [17], [18], [21]. Overexpression of *MIF* in macrophages induces arachidonic acid metabolism and COX-2 expression [37]. Increased expression of COX-2 and arachidonic acid are essential for inhibition of p53 activity by MIF [37]. This study shows upregulated MIF activity can be independent of COX-2-mediated arachidonic metabolism for induction of p53 activity. These interpretations do not rule out the possibility of non-reversible secondary messages that may have been triggered on account of the irreversible changes in gene expression leading to increased secretion of MIF in COX-2^−/−^ cells. Our observations negate the possible modulation of PGs and their influence on MIF. Studies on PGs have congruently recognized the prostaglandin-independent role of COXs in pathophysiology [13], [14], [15]. For example, esophageal squamous-cell carcinoma was inhibited by NSAIDs in COX-independent and dependent mechanism [38]. Thus, in this study the levels of eicosanoids have no significant role in the regulation of MIF in COX-2^−/−^ fibroblasts.

### MIF shares functional pathways with COX-2 and COX-1 independent of eicosanoid metabolism

The GO analysis shows distinct biological processes overlapping between MIF and COX-1 and COX-2 ablated cells. There seems to be functional compartmentalization of biological processes utilized by MIF in conjunction with COX-1 and COX-2 expression. MIF is known to promote leukocyte recruitment to sites of inflammation and during innate immune response [16], [17], [18], [19], [20], consistent with the observation in the GO analysis for molecular functions. Further examination of COX-2^−/−^ cells by GO molecular function analyses showed an increase in isomerase activity that included FK506 binding proteins (FKBPs) and triosephosphate isomerase (TPI), which was associated with MIF [39]. FKBPs play an essential role in immunosuppression and protein remodeling [40],whereas TPI plays an essential role in glycolysis and energy production [41]. The effect of MIF on immunosuppression and graft rejection is well documented [17], [42]. Thus upregulation of MIF in COX-2^−/−^ cells exhibits previouslyunrecognized functions in the MIF–COX axis.

### Co-modulation of MIF, COX, and p53 in cancer

MIF (but not DDT) is highly overexpressed in cells derived from leukemia’s (MOLT-4, K-562, and HL-60) and Burkett’s lymphomas (Daudi and Raji) [18], [43]. Increased expression of MIF in glioblastomas and esophageal squamous-cell carcinoma showed adverse prognostic outcomes during chemotherapy [43]. Also, MIF also promotes tumor growth and metastasis [16], [18], [23], [24], since MIF can mobilize the myeloid-derived suppressor cells (MDSCs) in the tumor microenvironment, thus augmenting immune suppression by the tumors [42]. In contrast, pharmacological intervention/inhibition of MIF diminishes MDSC buildup in the tumor [42]. Moreover, genetic deletion of MIF leads to decreased angiogenesis and inhibition of cell cycle as well as upregulation of p53 during reduced tumor burden [16], [18], [38], [44]. p53 works through several complex mechanisms in the modulation of cancer, including interactions with COX-2 and MIF [7], [18], [37], [38], [44].

p53 and COX-2 reciprocally regulate each other during inflammation and carcinogenesis [7], [11], [38], [44]. MIF can induce hypoxia in a p53-dependent manner, which can promote tumorigenesis [45]. Han et al. [46] have shown that p53-induced activity and apoptosis was significantly augmented in COX-2-null cells, but not wild-type cells. Subbaramaiah et al. [11] demonstrated that p53 suppressed the expression of *COX-2*, while a mutation in *TP53* led to increased basal COX-2 activity. Interestingly, p53 can bind to the −50 to +50 region in the TATA box of *COX-2* gene [11]. Our studies show that disruption of a *COX-2* gene induced oncogene and also p53 activity via bioinformatics analysis. The association between COX-1 and p53 further supports the over-activation of pro-oncogenic pathways in COX-2^−/−^ cells. Similar to COX-2 [12], the possibility that COX-1 may be able to interact directly via a protein–protein interaction with p53 remains promising. Overexpression of MIF modulates the functions of p53 and augments proinflammatory activity by physically interacting with p53 [44], [47].

Our bioinformatics analyses showed that loss of COX-2 activity and amplification of MIF is associated with transformed cells and brain tumors such as glioblastomas. The selective inhibitor of COX-2, NS-398 not only augmented the expression of MIF but induced differentiation of cancer cells [48]. Although there was activation of several functional pro-oncogenic pathways of p53 in COX-2^−/−^ > COX-1^−/−^ > WT + IL-1β > WT, the upregulation of MIF was observed only in COX-2^−/−^ cells. This observation further separates the phenotypic characteristics of COX-2^−/−^ cells from the others.

**Figure 7:**
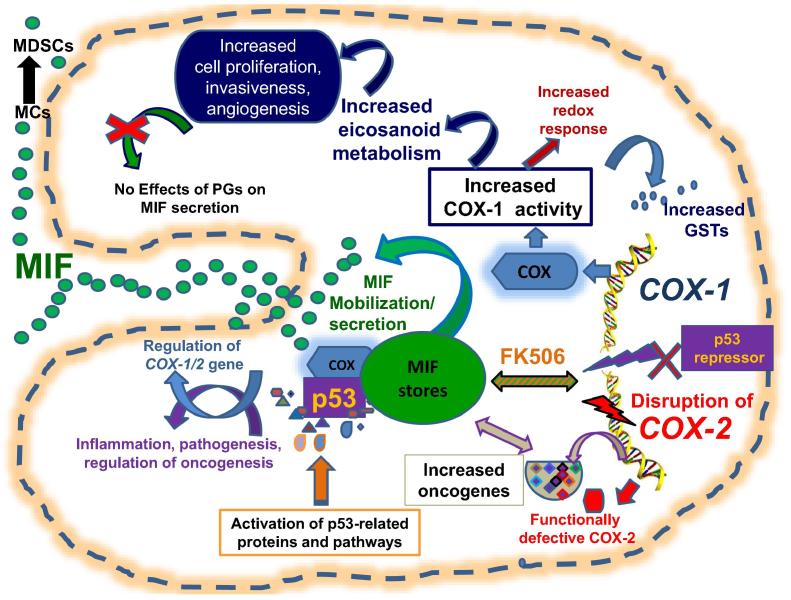
**A possible mechanism of action of the MIF–COX–p53 axis in COX-2^−/−^ cells** Cessation of *COX-2* activity led to upregulation of COX-1 gene expression and augmentation of COX-1 mediated eicosanoid productions, which are known to participate in inflammation, and other cellular activities such as cell proliferation, invasiveness, angiogenesis, and apoptosis [3], [4], [5], [6], [7], [8], [9]. Disruption of *COX-2* gene also induced constitutive production of MIF from performed intracellular stores independent of the levels of PGE_2_. MIF and COX activity may also participate in an FK506-related activity. The COX-2^−/−^ fibroblasts exhibit pro-oncogenic activity as evidenced by increased levels of transcripts of oncogenes, redox response elements, and p53-related activity. The regulation of MIF, p53, and COX-2 are linked. MIF and p53 can bind via their cysteine residues C81 and C242, respectively [47]. The complex between p53 and its negative regulators MDM2 can be stabilized by MIF [1], [2], [18]. p53 is known to suppress COX-2 gene expression by occupying the TATA box in the COX-2 promoter [11] or p53 can induce COX-2 to counterbalance stress-induced apoptosis [46]. Increased activity of MIF and COX-1 is also allied to the development of brain tumors and colon adenomas [6], [7], [18], [43]. The cross-talk between COX and p53 plays a balancing role during inflammatory stress and carcinogenesis [6], [7], [11], [12], [62]. MIF also promotes carcinogenesis by stimulating the conversion of myeloid cells to myeloid-derived suppressor cells within the tumor, which protect the tumor from immune response [42]. The MIF–COX–p53 axis is involved in cellular homeostasis, sterile inflammation, and oncogenesis. The COX-2^−/−^ cells exhibit a proinflammatory and pro-oncogenic signature at the molecular level, which encompasses three pleiotropic pathways of COX, MIF, and p53 [9], [18], [43]. MDM2, mouse double minute 2 homolog; MC, myeloid cell; MDSC, myeloid-derived suppressor cell; GST, glutathione *S*-transferase.

In summary, these studies demonstrate a notable crosstalk between MIF-COX axis, p53, and other oncogenes. These signaling pathways cooperate to strike a delicate balance between cell cycle, senescence, death and during COX-1 induced chronic inflammation (Figure 7). Inhibition of MIF expression (by anti-MIF or anti-CD74 antibodies or chemotherapy) or COX expression (by indomethacin) has shown encouraging results, which may hinge upon the type of cancer [24]. Given the compensation of eicosanoid production by COX-1 pathway [4], the possibility of utilizing nonspecific COX [4], [6], [10] plus MIF [16], [18], [26] inhibitors for cancer prevention and therapy cannot be ruled out.

## Conclusion

The genetic impairment of *COX-2* augments *COX-1* expression and PG production, generating a proinflammatory state within the fibroblasts. This genetic change also triggers several PG-independent functions such as the constitutive production of MIF. Both MIF and COX share functions outside of eicosanoid metabolism. The COX-2^−/−^ cells also exhibit an increased activity of several oncogenes including p53-related modulators, which tilts the COX-2^−/−^ cells toward a pro-oncogenic state. Consequently, the coordinated COX-MIF-p53 axis regulates an intracellular environment of inflammation and oncogenesis.

## Materials and methods

All the media, growth factors, and fetal calf serum (FCS) for cell culture were procured from Gibco BRL (Life Technologies, Carlsbad, CA). Gel Code Silver SNAP Staining Kit was obtained from Pierce (Rockford, IL). The ELISA kit for detection of MIF was purchased from Chemicon International (Temecula, CA) and anti-MIF antibodies were procured from Santa Cruz Biotech (Santa Cruz, CA).

### Culture of COX-deficient mouse lung fibroblasts

Lung fibroblasts (10^5^ cells/ml) were procured from wild-type C57BL/6J, COX-1^−/−^, and COX-2^−/−^ mice as previously reported [49], [50]. These fibroblasts were seeded in DMEM containing high glucose, antibiotics, and 10% FCS at 37 °C in 5% CO_2_ incubator for 24 h until the cells were confluent [4], [49], [50]. In selected experiments, the cells were incubated with 10 ng/ml of IL-1β as described in the legends.

### Immunoblotting

Total cytosolic protein extracts of COX-1^−/−^, COX-2^−/−^, and wild-type fibroblast cells were generated as described previously [4], [28] using the Pierce protein extraction kit (Pierce, Rockford, IL). 10–100 μg of total cytosolic lysate from each cell type was separated on 12% SDS–PAGE gels. Proteins on the gels were visualized by Coomassie Brilliant Blue R 250 or silver staining. If required, the separated proteins were transferred onto a nitrocellulose membrane for immunoblotting and subjected to the reversible Ponceau S staining for loading quantification. After blocking with 3% BSA, membranes were incubated with anti-MIF antibodies (1:2000) as recommended by the manufacturer (INOVUS Biologics, Littleton, CO) followed by peroxidase-labeled secondary antibodies. Proteins were visualized using the chemiluminescent detection system (ECL Plus, Amersham, Arlington Heights, IL).

### Amino acid sequence analysis

The cytosolic fraction of the protein extract from COX-2^−/−^ fibroblast cells was separated on a long 15% SDS–PAGE gel and transferred onto Immobilon P membranes (Millipore, Bedford, MA, USA). After staining with Coomassie blue R-250, the 10−15 kDa protein band(s) were excised from the Immobilon P membrane and subjected to automated N-terminal Edman degradation using Procise Protein Sequencer (Applied Biosystems, Carlsbad, CA, USA) following standard protocols. The N-terminal sequence was searched for homologs in the Swiss-Prot and TrEMBL databases (http://us.expasy.org) using the ScanProsite algorithm [51], [52].

### ELISA analysis

Equal amounts of wild-type, COX-1^−/−^, and COX-2^−/−^ fibroblast cells were grown as described above and the cell supernatants were analyzed using a capture ELISA kit (Chemikine™) to detect the mouse MIF protein as instructed by the manufacturer (Chemicon International, Temecula, CA).

### Analysis of PGs and MIF

The wild-type, COX-1^−/−^, and COX-2^−/−^ cells were grown as described above and the secretion of PGE_2_ in the medium was estimated using a radioimmunoassay (RIA) at 24 h as previously reported [4], [28]. Cells were also treated with 5 µM of indomethacin or 0.5 µM of arachidonic acid for 24 h from time zero, to monitor levels of PGE_2_ and MIF_._ The amount of MIF was estimated by ELISA as described above.

### Labeling and hybridization of microarray gene chips

The wild-type, COX-1^−/−^, and COX-2^−/−^ fibroblast cells were grown for 24 h as described above. Total RNA extraction and cDNA synthesis were performed using Invitrogen SuperScript (Invitrogen, Carlsbad, CA) as previously reported [4]. Biotin-labeled cDNA was produced using ENZO BioArray High Yield RNA transcript labeling kit (Affymetrix, Santa Clara, CA). The labeled cDNA was purified utilizing a Qiagen RNeasy kit. The cDNA was fragmented at 95 °C for 35 min for target preparation. The Murine Genome Array U74Av2 Array (Affymetrix) was used for gene expression array analysis [4].

### Normalization and analysis of the microarray data

The test samples (from WT, COX-1^−/−^, COX-2^−/−^ cells, and IL-1β treated WT cells) were hybridized to different probes on the gene chip and subjected to an Affymetrix scanner for signal normalization and quantification [4]. Replicate samples were processed for the COX-1^−/−^, COX-2^−/−^, and IL-1β-treated WT cells. Microarray data were normalized as previously reported [4]. The Affymetrix.cel microarray files (U74Av2 arrays) from all experimental conditions (COX-1^−/−^, COX-2^−/−^, WT, and IL-1β-stimulated WT cells) and all replicates were normalized together (within the array and between array) in order to compare gene expression of each gene/transcript across all experimental conditions. The robust multi-array average (RMA) method, which is available in Bioconductor “Affy” and “Limma” package, was used for normalization using the default parameter as described (https://www.bioconductor.org/).

Probes were annotated to genes. The average expression levels were utilized when more than one probe corresponded to the same gene. Four housekeeping genes (*GAPDH*, *β actin*, *RPL30*, *RPS13*) [32], showed similar expression of basal levels in WT, COX-1^−/−^, COX-2^−/−^ cells and IL-1β treated WT cells. The mean value of all the four housekeeping genes from the gene expression array was taken as basal value, and the data were presented as arbitrary units (AU) as previously reported [4]. Fold changes (FCs) in gene expression were computed by averaging the logged signal values for the replicate samples after comparing them with WT. Genes with FC ≥ 1.75 (upregulated) or ≤0.5 (downregulated) [53], [54] are defined as differentially expressed genes (DEGs).

### GO analysis

All DEGs in WT + IL-1β, COX-1^−/−^, COX-2^−/−^ were extracted from [4] (Figures S1–S4). Functional annotation of the DEGs was performed based on Gene Ontology Consortium 2000 (http://www.geneontology.org) [55], [56] and KEGG pathway database [57]. Genes are classified according to GO biological process and KEGG pathways. The GO biological process/pathway categories containing ≥10 annotated genes were retained for the enrichment analysis, and heat maps were generated using Gitools (www.gitools.org) [58], [59]. The resulting *P* values were adjusted for multiple testing using the Benjamin–Hochberg method of false discovery rate (FDR) [60]. A detail bioinformatics method for GO analysis and enrichment analysis is described in File S1.

Similarly, for the enrichment analysis of MIF-related pathways, we considered the GO terms or KEGG pathway having *MIF* present as one of the component genes. For enrichment analysis [56], *TP53* target genes that directly interact with p53 in various p53 pathways were used for the study (Table S1). These pathways are described at the TP53 Web Site (https://p53.fr/tp53-information/tp53-knowledge-center/26-knowledge-center/28-p53-pathways).

### Cancer association analysis

We searched the IntOGen database (www.intogen.org) [59] to identify different types of cancers, in which expression of COX-2 and MIF was significantly up-regulated or downregulated, or lost or gained and extracted the data using Biomart data extraction facility [58]. Cancer types and their respective *P*-value of significance were listed in Tables S2 and S3. The detail bioinformatics procedure is described in File S2.

### Hierarchical clustering (HCL) and heatmaps

Hierarchical clustering analysis was performed using MeV (Multiple Experiment Viewer) of TM4 suit [61] with Euclidean distance and average linkage.

### Statistical analysis

A GraphPad Software (V1.14) (San Diego, CA) was used for statistical analyses as previously reported [54]. Data were analyzed using the Wilcoxon–Mann–Whitney test where applicable and represented as the mean ± standard deviation (*n* ≥ 3 samples). For all the tests, the difference with *P* < 0.05 was considered significant.

## Authors’ contributions

The project was conceived by ARA. AI led the bioinformatics analyses with RVJ. MD was involved in the MIF and PGE_2_ analysis. MD, AR, and JG were involved in protein sequencing. All authors participated in the manuscript preparation, read and approved the final manuscript.

## Competing interests

The project was supported by a research contract involving Target Discovery, Validation, and Functional Genomics from Yamanuchi Pharmaceuticals [Astellas] which covered part of ARA’s and MD’s salaries.

## Supplementary material

Supplementary Figure S1 Functional analysis of increased *MIF* expression in COX-1^−/−^, COX-2^−/−^, and IL-1β-treated WT cells. Heatmap shows upregulated *MIF* gene expression for GO modules with MIF as one of the components. MIF expression in COX-1^−/−^, or COX-2^−/−^ and L-1β-treated WT cells is compared, using MIF expression in WT as the background control A. Biological process. B. Molecular function. C. Expanded cluster of isomerases with upregulated *MIF* expression in COX-2^−/−^ cells.

Supplementary Figure S2 Relative gene expression of *MIF*, *COX-1*, and *COX-2* in various cell groups. WT, WT + IL-1β, COX-1^−/−^, and COX-2^−/−^ cells were subjected to gene expression array [4]. The mRNA expression levels were plotted in arbitrary units as a ratio of WT, after normalizing with the average expression levels of four housekeeping genes including *Actin*, *GAPDH*, *RPL30*, and *RPS13*. Expression in the form of heat map (marked as arrow) as observed on the gene expression arrays using Gitools is presented on the left [4].

Supplementary Figure S3 Murine *MIF-Ddt-Gstt* gene cluster on chromosome 10. Genes encoding macrophage inhibitory factor (*MIF*)*,* D-dopachrome tautomerase (*Ddt*), glutathione *S*-transferase theta 1 (*Gstt1*), and glutathione *S*-transferase, theta 2 (*Gstt2*) are located closely as a cluster on Chromosome 10 in mice.

Supplementary Figure S4 Differential expression of *PTGS1*, *PTGS2*, and *TP53* in cancers. The heatmap shows mRNA expression for *TP53* (p53)*, PTGS1* (COX-1), and *PTGS2* (COX-2) in the tumors. The heatmap was generated from IntOGen with the corrected *P* value (FDR) of significance indicated.

Supplementary Table S1 Modulation of p53-related transcripts and regulatory pathways in WT, COX-1^−/−^, COX-2^−/−^, and IL-1β-treated WT cells.

Supplementary Table S2 Expression of COX-2 in various cancers.

Supplementary Table S3. Tumors with loss of COX-2 and gain of MIF

File S1 GO analysis and enrichment analysis of biological functions.

File S2 Extraction and bioinformatic analysis of cancer.
