# Supplementary material for: Proteomic Analysis Shows Constitutive Secretion of MIF and p53-associated Activity of COX-2−/− Lung Fibroblasts
Source: Genomics Proteomics Bioinformatics. 2017 Dec 13;15(6):339–51. doi: 10.1016/j.gpb.2017.03.005 (PMC5828655; doi:10.1016/j.gpb.2017.03.005)
Supplement: Supplementary Figure S1 — Functional analysis of increased MIF expression in COX-1−/−, COX-2−/−, and IL-1β-treated WT cells. Heatmap shows upregulated MIF gene expression for GO modules with MIF as one of the components. MIF expression in COX-1−/−, or COX-2−/− and L-1β-treated WT cells is compared, using MIF expression in WT as the background control A. Biological process. B. Molecular function. C. Expanded cluster of isomerases with upregulated MIF expression in COX-2−/− cells. [file mmc1.pptx]

## Slide 1
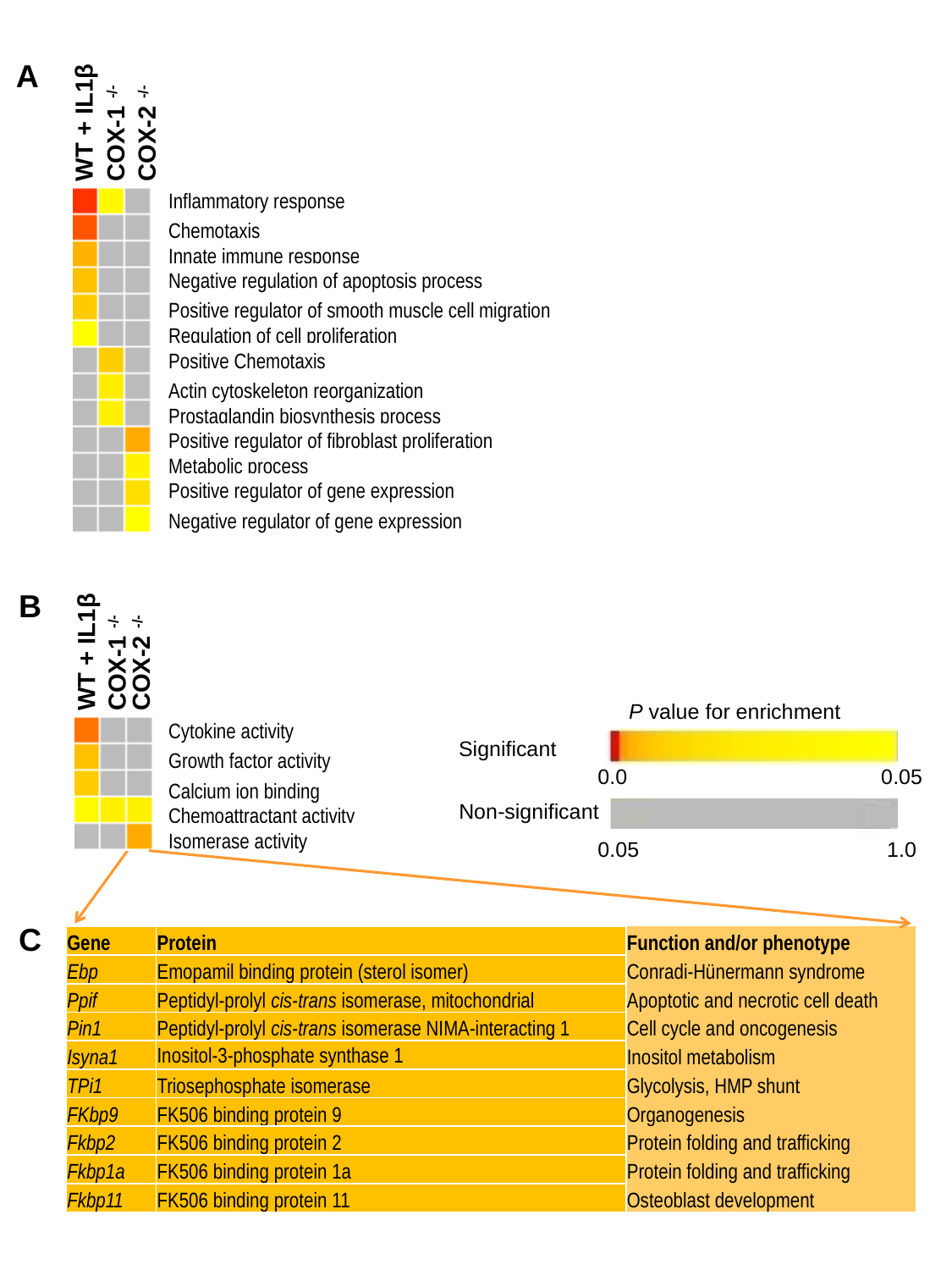

A
WT + IL1β
COX-1 -/-
COX-2 -/-
Inflammatory response
Chemotaxis
Innate immune response
Negative regulation of apoptosis process
Positive regulator of smooth muscle cell migration
Regulation of cell proliferation
Positive Chemotaxis
Actin cytoskeleton reorganization
Prostaglandin biosynthesis process
Positive regulator of fibroblast proliferation
Metabolic process
Positive regulator of gene expression
Negative regulator of gene expression
B
WT + IL1β
COX-1 -/-
COX-2 -/-
Cytokine activity
Growth factor activity
Calcium ion binding
Chemoattractant activity
Isomerase activity
Significant
0.0 0.05
00
P value for enrichment
Non-significant
0.05 1.0
C
| Gene | Protein | Function and/or phenotype |
| --- | --- | --- |
| Ebp | Emopamil binding protein (sterol isomer) | Conradi-Hünermann syndrome |
| Ppif | Peptidyl-prolyl cis-trans isomerase, mitochondrial | Apoptotic and necrotic cell death |
| Pin1 | Peptidyl-prolyl cis-trans isomerase NIMA-interacting 1 | Cell cycle and oncogenesis |
| Isyna1 | Inositol-3-phosphate synthase 1 | Inositol metabolism |
| TPi1 | Triosephosphate isomerase | Glycolysis, HMP shunt |
| FKbp9 | FK506 binding protein 9 | Organogenesis |
| Fkbp2 | FK506 binding protein 2 | Protein folding and trafficking |
| Fkbp1a | FK506 binding protein 1a | Protein folding and trafficking |
| Fkbp11 | FK506 binding protein 11 | Osteoblast development |
