# Supplementary material for: Proteomic Analysis Shows Constitutive Secretion of MIF and p53-associated Activity of COX-2−/− Lung Fibroblasts
Source: Genomics Proteomics Bioinformatics. 2017 Dec 13;15(6):339–51. doi: 10.1016/j.gpb.2017.03.005 (PMC5828655; doi:10.1016/j.gpb.2017.03.005)
Supplement: Supplementary Figure S2 — Relative gene expression of MIF, COX-1, and COX-2 in various cell groups. WT, WT + IL-1β, COX-1−/−, and COX-2−/− cells were subjected to gene expression array [4]. The mRNA expression levels were plotted in arbitrary units as a ratio of WT, after normalizing with the average expression levels of four housekeeping genes including Actin, GAPDH, RPL30, and RPS13. Expression in the form of heat map (marked as arrow) as observed on the gene expression arrays using Gitools is presented on the left [4]. [file mmc2.ppt]

## Slide 1
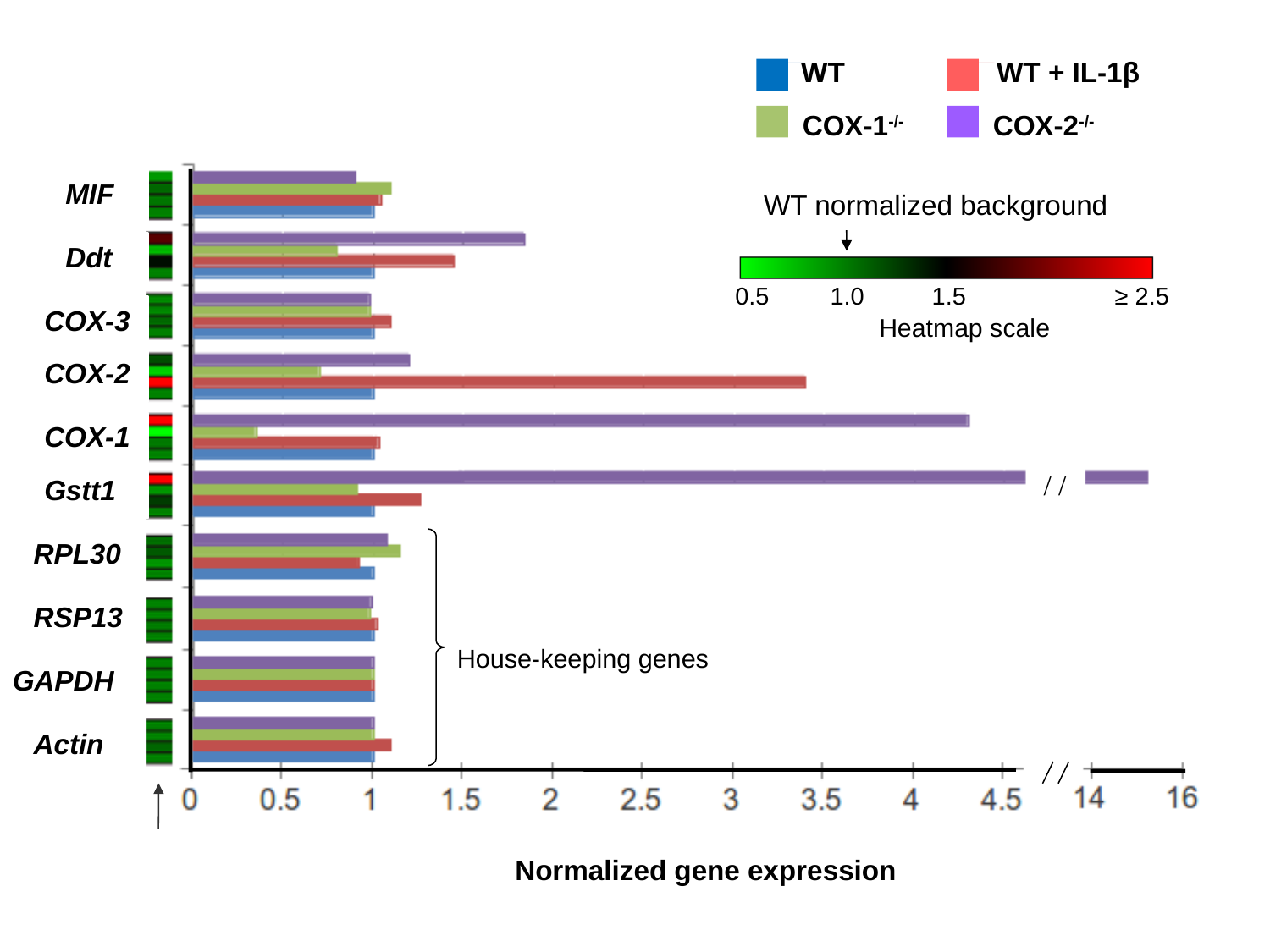

WT
WT + IL-1β
COX-1-/-
COX-2-/-
WT normalized background
 0.5 1.0 1.5 ≥ 2.5
Heatmap scale
MIF
Ddt
COX-3
COX-2
COX-1
/ /
Gstt1
RPL30
RSP13
House-keeping genes
GAPDH
Actin
Normalized gene expression
