# Supplementary figures and images for: Proteomic Analysis Shows Constitutive Secretion of MIF and p53-associated Activity of COX-2−/− Lung Fibroblasts
Source: Genomics Proteomics Bioinformatics. 2017 Dec 13;15(6):339–51. doi: 10.1016/j.gpb.2017.03.005 (PMC5828655; doi:10.1016/j.gpb.2017.03.005)

## Slide 1
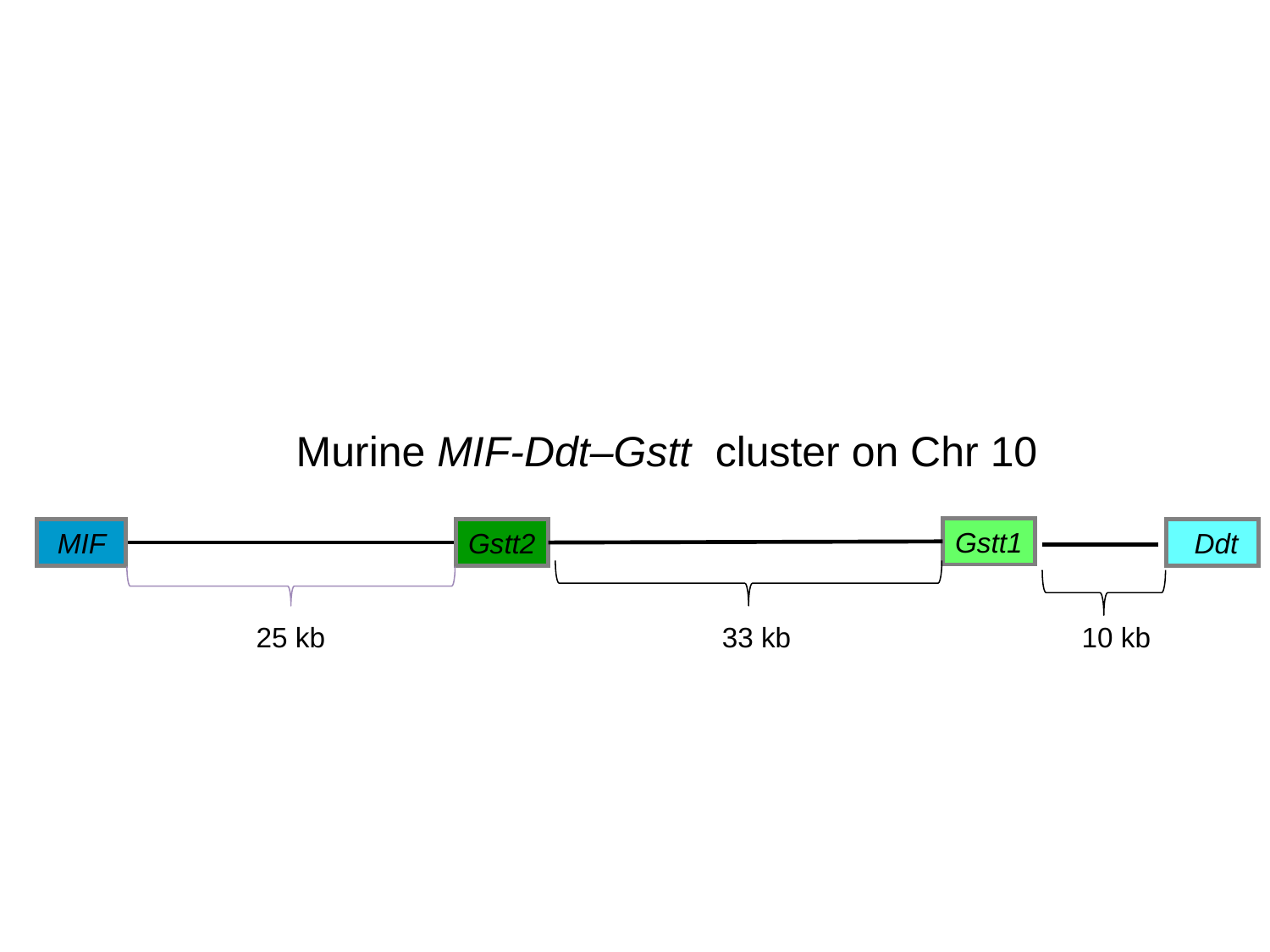

Murine MIF-Ddt–Gstt cluster on Chr 10
Gstt1
 MIF
Gstt2
 Ddt
25 kb
33 kb
10 kb

Supplement: Supplementary Figure S3 — Murine MIF-Ddt-Gstt gene cluster on chromosome 10. Genes encoding macrophage inhibitory factor (MIF), D-dopachrome tautomerase (Ddt), glutathione S-transferase theta 1 (Gstt1), and glutathione S-transferase, theta 2 (Gstt2) are located closely as a cluster on Chromosome 10 in mice. [file mmc3.pptx]

## Slide 1
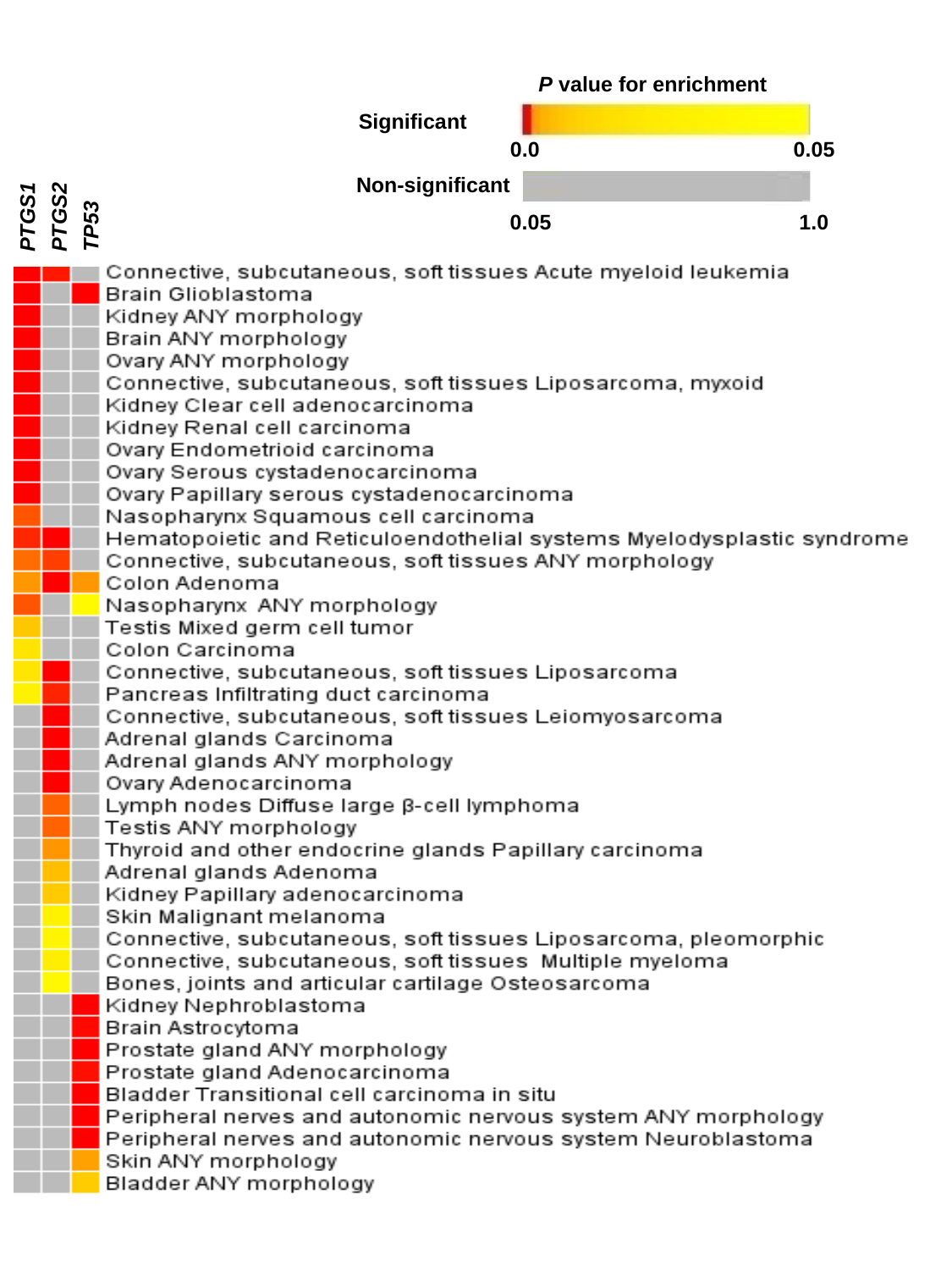

Significant
0.0 0.05
00
P value for enrichment
Non-significant
0.05 1.0
PTGS1
PTGS2
TP53

Supplement: Supplementary Figure S4 — Differential expression of PTGS1, PTGS2, and TP53 in cancers. The heatmap shows mRNA expression for TP53 (p53), PTGS1 (COX-1), and PTGS2 (COX-2) in the tumors. The heatmap was generated from IntOGen with the corrected P value (FDR) of significance indicated. [file mmc4.pptx]
