# Supplementary material for: Proteomic Analysis Shows Constitutive Secretion of MIF and p53-associated Activity of COX-2−/− Lung Fibroblasts
Source: Genomics Proteomics Bioinformatics. 2017 Dec 13;15(6):339–51. doi: 10.1016/j.gpb.2017.03.005 (PMC5828655; doi:10.1016/j.gpb.2017.03.005)
Supplement: Supplementary Table S1 — Modulation of p53-related transcripts and regulatory pathways in WT, COX-1−/−, COX-2−/−, and IL-1β-treated WT cells. [file mmc5.docx]

**Table S1 Modulation of p53-related transcripts and regulatory pathways in WT, COX-1^-/-^, COX-2^-/-^, and IL-1β-treated WT cells**

| **Gene symbol** | **Protein name** | **WT** | **FC (IL1β/ WT)** | **FC (COX-1^-/-^/WT)** | **FC (COX-2^-/-^/WT)** | **Pathways related to p53** |
| --- | --- | --- | --- | --- | --- | --- |
| *Xpc* | Xeroderma pigmentosum, complementation group C | 10.137 | 1.4765 | 1.7772 | 1.2598 | p53 and DNA repair |
| *Usp4* | Ubiquitin specific peptidase 4 (proto-oncogene) | 13.092 | 0.7274 | 0.6887 | 0.4861 | p53 antagonist |
| *Usp22* | Ubiquitin specific peptidase 22 | 9.871 | 1.5618 | 1.5094 | 1.8303 | p53 antagonist |
| *Urm1* | Ubiquitin related Modifier [SUMO] | 11.964 | 1.1506 | 1.2813 | 1.3539 | Co-regulated with p53 |
| *Ubr5* | Ubiquitin protein ligase E3 component n-recognin 5 | 8.759 | 1.8005 | 1.6060 | 1.8624 | Co-regulated with p53 |
| *Ube2n* | Ubiquitin-conjugating enzyme E2N | 12.760 | 1.5350 | 1.7551 | 2.4069 | Co-regulated with p53 |
| *Ube2m* | Ubiquitin-conjugating enzyme E2M (UBC12 homolog, yeast) | 12.074 | 1.0733 | 1.0410 | 1.2988 | p53 and DNA repair |
| *Ube2e1* | Ubiquitin-conjugating enzyme E2E 1, UBC4/5 homolog (yeast) | 10.618 | 1.0243 | 2.3476 | 2.9940 | p53 interacting enzyme |
| *Ube2d3* | Ubiquitin-conjugating enzyme E2D 3 (UBC4/5 homolog, yeast) | 11.190 | 0.8540 | 0.9358 | 0.3843 | Co-regulated with p53 |
| *Ubc* | Ubiquitin C | 15.201 | 1.1229 | 1.0591 | 1.0520 | Co-regulated with p53 |
| *Ubac2* | Ubiquitin associated domain containing 2 | 8.754 | 1.3586 | 1.7063 | 1.7844 | Co-regulated with p53 |
| *Tpd52l1* | Tumor protein D52-like 1 | 11.486 | 0.9664 | 0.7289 | 2.6345 | Co-regulated with p53 |
| *Tpd52* | Tumor protein D52 | 8.945 | 1.5579 | 1.7151 | 1.4349 | Co-regulated with p53 |
| *Tnfaip1* | Tumor necrosis factor, alpha-induced protein 1 (endothelial) | 9.692 | 1.8807 | 2.0635 | 2.2924 | Co-regulated with p53 |
| *Tbp* | TATA box binding protein | 10.694 | 1.0283 | 1.1528 | 1.2459 | Co-modulator of transcription |
| *St13* | Suppression of tumor 13 | 11.960 | 1.2907 | 0.9906 | 1.8643 | p53 and DNA repair |
| *Smu1* | Smu-1 suppressor of *MES*-8 and unc-52 homolog (C. elegans) | 11.904 | 1.5911 | 1.2645 | 1.4846 | Co-regulated with p53 |
| *Rsu1* | Ras suppressor protein 1 | 12.861 | 0.9335 | 1.3531 | 1.3152 | Co-regulated with p53 |
| *Rbbp7* | Retinoblastoma binding protein 7 | 13.680 | 1.0849 | 1.3496 | 1.4338 | Co-regulated with p53 |
| *Rb1* | Retinoblastoma 1 | 7.164 | 1.2512 | 1.1792 | 1.2180 | Co-regulated with p53 |
| *Rasa1* | RAS p21 protein activator 1 [Ras p21] | 11.158 | 0.6903 | 1.2096 | 0.4392 | Co-regulated with p53 |
| *Ptov1* | Prostate tumor over expressed gene 1 | 11.751 | 0.9636 | 1.6314 | 1.9396 | Co-regulated with p53 |
| *Prkdc* | Protein kinase, DNA activated, catalytic polypeptide | 10.431 | 0.6870 | 0.5997 | 0.5954 | Co-regulated with p53 |
| *Prkcq* | Protein kinase C, theta | 8.419 | 1.4673 | 1.4272 | 1.5384 | Co-regulated with p53 |
| *Pmp22* | Peripheral myelin protein 22 | 12.163 | 0.9930 | 0.7019 | 0.2610 | p53 and apoptosis |
| *Nub1* | Negative regulator of ubiquitin-like proteins 1 | 11.839 | 0.9128 | 0.8907 | 0.5498 | Translocating p53 |
| *Nbl1* | Neuroblastoma, suppression of tumor 1 | 13.540 | 1.5233 | 0.7485 | 0.5587 | Co-regulated with p53 |
| *Myc* | Myelocytomatosis oncogene | 11.324 | 1.0174 | 0.9721 | 2.7587 | Co-regulated with p53 |
| *Mdm2* | Transformed mouse 3T3 cell double minute 2 | 14.934 | 0.8325 | 1.0997 | 6.1828 | p53 antagonist |
| *Lats2* | Large tumor suppressor 2 | 12.463 | 1.0010 | 2.0804 | 1.9379 | Co-regulated with p53 |
| *Gtse1* | G two S phase expressed protein 1 | 12.080 | 1.1590 | 1.0896 | 1.2708 | Cell cycle/division |
| *Gm9706* | Predicted gene 9706 / ISG15 ubiquitin-like modifier | 11.906 | 0.9213 | 0.3721 | 0.1361 | p53 interacting enzyme |
| *Gm5801* | Ubiquitin-conjugating enzyme E2, J2 homolog pseudogene / | 11.823 | 1.0717 | 0.9773 | 1.2850 | p53 interacting enzyme |
| *Gadd45a* | Growth arrest and DNA-damage-inducible 45 alpha | 12.089 | 2.1075 | 2.2974 | 2.3764 | p53 and DNA repair |
| *Fas* | Fas (TNF receptor superfamily member 6) | 9.462 | 3.3863 | 1.1546 | 1.4828 | p53 and apoptosis |
| *E2f1* | E2F transcription factor 1 | 10.478 | 1.1043 | 1.6538 | 1.2405 | Co-modulator of transcription |
| *Csnk1a1* | Casein kinase 1, alpha 1 | 12.674 | 0.9494 | 0.7425 | 0.4653 | Co-regulated with p53 |
| *Cited2* | Cbp/p300-interacting transactivation, | 13.882 | 0.8425 | 1.1810 | 3.0990 | Co-regulated with p53 |
| *Cenpa* | Centromere protein A | 11.315 | 1.3987 | 1.6436 | 1.9470 | Co-regulated with p53 |
| *Cdkn3* | Cyclin-dependent kinase inhibitor 3 | 11.690 | 1.0045 | 1.0699 | 1.8969 | p53 and DNA repair |
| *Cdkn2b* | Cyclin-dependent kinase inhibitor 2B (p15, inhibits CDK4) | 11.164 | 0.9568 | 0.7889 | 5.7257 | Cell cycle/division |
| *Cdkn2a* | Cyclin-dependent kinase inhibitor 2A | 11.632 | 1.1472 | 1.1730 | 1.8876 | Cell cycle/division |
| *Cdk1* | Cyclin-dependent kinase 1 | 13.539 | 1.3558 | 1.8688 | 2.8406 | Cell cycle/division |
| *Cdca8* | Cell division cycle associated 8 | 11.298 | 1.4093 | 1.1988 | 2.5607 | Cell cycle/division |
| *Cdca3* | Cell division cycle associated 3 | 11.199 | 1.1213 | 1.0314 | 1.4117 | Cell cycle/division |
| *Cdc7* | Cell division cycle 7 (S. cerevisiae) | 10.616 | 1.5493 | 2.5935 | 1.8031 | Cell cycle/division |
| *Ccng1* | Cyclin G1 | 10.683 | 1.1571 | 1.7802 | 1.6786 | Cell cycle/division |
| *Ccnf* | Cyclin F | 11.464 | 1.3782 | 1.4301 | 1.5427 | p53 antagonist |
| *Ccnd1* | Cyclin D1 | 13.144 | 0.8394 | 2.7857 | 2.7938 | Cell cycle/division |
| *Ccnb2* | Cyclin B2 | 14.005 | 0.8889 | 0.9982 | 1.9912 | Cell cycle/division |
| *Ccar1* | Cell division cycle and apoptosis regulator 1 | 9.333 | 1.4466 | 1.4837 | 1.5432 | p53 and apoptosis |
| *Bax* | BCL2-associated X protein | 13.274 | 1.4034 | 1.3079 | 2.1873 | Co-modulator of transcription |
| *Atm* | Ataxia telangiectasia mutated homolog (human) | 10.300 | 1.0235 | 0.8633 | 0.6089 | Cell cycle/division |
| *Arih1* | Ubiquitin-conjugating enzyme E2 binding protein | 11.534 | 0.8421 | 0.8831 | 0.4370 | p53 interacting enzyme |
| *Apex1* | Apurinic/apyrimidinic endonuclease 1 | 12.819 | 1.0835 | 1.5014 | 2.2174 | Cell cycle/division |
| *Abcb1b* | ATP-binding cassette, sub-family B (MDR/TAP), member 1B | 8.822 | 0.4645 | 0.5787 | 0.5908 | Co-regulated with p53 |

*Note*: WT represents the basal expression for each transcript and expression is expressed in log2.
