# Supplementary material for: Proteomic Analysis Shows Constitutive Secretion of MIF and p53-associated Activity of COX-2−/− Lung Fibroblasts
Source: Genomics Proteomics Bioinformatics. 2017 Dec 13;15(6):339–51. doi: 10.1016/j.gpb.2017.03.005 (PMC5828655; doi:10.1016/j.gpb.2017.03.005)
Supplement: Supplementary Table S2 — Expression of COX-2 in various cancers. [file mmc6.docx]

**Table S2 Expression of COX-2 in various tumors**

| **Topography and morphology** | ***P* value for COX-2 upregulation** | ***P* value for COX-2 downregulation** |
| --- | --- | --- |
| Brain [C71]; ANY morphology | 1.000 | 5.37E-13 |
| Brain [C71]; glioblastoma, nos [9440/3] | 1.000 | 7.74E-11 |
| Adrenal glands [C74]; adenoma, nos [8140/0] | 5.00E-03 | 0.002 |
| Adrenal glands [C74]; ANY morphology | 2.09E-07 | 1.21E-05 |
| Adrenal glands [C74]; carcinoma, nos [8010/3] | 1.00E-06 | 0.033 |
| Bladder [C67]; transitional cell carcinoma in situ [8120/2] | 1.000 | 1.00E-06 |
| Kidney [C64]; nephroblastoma, nos [8960/3] | 0.052 | 1.00E-06 |
| Kidney [C64]; papillary adenocarcinoma, nos [8260/3] | 0.007 | 0.007 |
| Kidney [C64]; renal cell carcinoma, nos [8312/3] | 0.628 | 1.00E-06 |
| Liver and hepatic bile ducts [C22]; hepatocellular adenoma, nos [8170/0] | 0.943 | 1.000 |
| Lymph nodes [C77]; malignant lymphoma, t-cell nos [9702/3] | 1.000 | 1.000 |
| Ovary [C56]; ANY morphology | 1.000 | 4.86E-12 |
| Ovary [C56]; endometrioid carcinoma, nos [8380/3] | 1.000 | 1.00E-06 |
| Ovary [C56]; papillary serous cystadenocarcinoma [8460/3] | 1.000 | 1.00E-06 |
| Ovary [C56]; serous cystadenocarcinoma, nos [8441/3] | 1.000 | 1.38E-07 |
| Peripheral nerves and autonomic nervous system [C47]; ANY morphology | 1.000 | 1.00E-06 |
| Peripheral nerves and autonomic nervous system [C47]; neuroblastoma, nos [9500/3] | 1.000 | 1.00E-06 |
| Skin [C44]; ANY morphology | 0.202 | 0.012 |
| Stomach [C16]; adenocarcinoma, nos [8140/3] | 1.000 | 0.003 |
| Stomach [C16]; ANY morphology | 1.000 | 0.001 |
| Testis [C62]; mixed germ cell tumor [9085/3] | 0.822 | 0 |
| Hematopoietic and reticuloendothelial systems [C42]; myelodysplastic syndrome, nos [9989/3] | 9.97E-07 | 0.902 |
| Hematopoietic and reticuloendothelial systems [C42]; ANY morphology | 4.73E-05 | 0.933 |
| Pancreas [C25]; infiltrating duct carcinoma, nos [8500/3] | 1.82E-05 | 1.000 |

*Note*: The data were generated from the IntOGen with the corrected *P* value (FDR) of significance included. The significant *P* values (< 0.05) are shaded in color.
