# Supplementary material for: Proteomic Analysis Shows Constitutive Secretion of MIF and p53-associated Activity of COX-2−/− Lung Fibroblasts
Source: Genomics Proteomics Bioinformatics. 2017 Dec 13;15(6):339–51. doi: 10.1016/j.gpb.2017.03.005 (PMC5828655; doi:10.1016/j.gpb.2017.03.005)
Supplement: Supplementary Table S3. — Tumors with loss of COX-2 and gain of MIF [file mmc7.docx]

**Table S3 Tumors with loss of COX-2 and gain of MIF**

| **Topography and morphology** | ***P* value for loss of COX-2** | ***P* value for gain of MIF** |
| --- | --- | --- |
| Brain [C71]; ependymoma, nos [9391/3] | 0.012 | 5.77E-005 |
| Spinal cord, cranial nerves and other parts of central nervous system [C72]; neuroblastoma, nos [9500/3] | 2.59E-007 | 0.00E+000 |
| Spinal cord, cranial nerves and other parts of central nervous system [C72]; ANY morphology | 5.62E-006 | 4.26E-012 |
| Adrenal glands [C74]; ANY morphology | 1.00E-006 | 0.244 |
| Adrenal glands [C74]; pheochromocytoma, nos [8700/0] | 1.00E-006 | 0.244 |
| Connective, subcutaneous and other soft tissues [C49]; synovial sarcoma, monophasic [9041/3] | 0.002 | 0.341 |
| Brain [C71]; choroid plexus papilloma, nos [9390/1] | 0.074 | 1.00E-006 |
| Brain [C71]; ANY morphology | 1.000 | 0.000 |
| Brain [C71]; glioblastoma, nos [9440/3] | 1.000 | 1.20E-013 |
| Brain [C71]; mixed glioma [9382/3] | 1.000 | 4.41E-006 |
| Colon [C18]; adenocarcinoma, nos [8140/3] | 1.000 | 1.84E-004 |
| Hematopoietic and reticuloendothelial systems [C42]; adult t-cell leukemia/lymphoma [htlv-1 pos.] [9827/3] | 0.544 | 1.77E-006 |
| Hematopoietic and reticuloendothelial systems [C42]; follicular lymphoma, nos [9690/3] | 0.977 | 8.49E-005 |
| Kidney [C64]; nephroblastoma, nos [8960/3] | 1.000 | 1.74E-004 |
| Kidney [C64]; renal cell carcinoma, nos [8312/3] | 0.997 | 3.39E-006 |
| Liver and hepatic bile ducts [C22]; ANY morphology | 1.000 | 0.001 |
| Lymph nodes [C77]; diffuse large b-cell lymphoma, nos [9680/3] | 1.000 | 0.007 |
| Lymph nodes [C77]; malignant lymphoma, t-cell nos [9702/3] | 0.494 | 1.32E-006 |
| Ovary [C56]; ANY morphology | 1.000 | 1.90E-004 |
| Pancreas [C25]; ANY morphology | 1.000 | 7.38E-005 |
| Peripheral nerves and autonomic nervous system [C47]; ANY morphology | 0.931 | 7.36E-009 |
| Peripheral nerves and autonomic nervous system [C47]; malignant peripheral nerve sheath tumor [9540/3] | 1.000 | 0.001 |
| Peripheral nerves and autonomic nervous system [C47]; neuroblastoma, nos [9500/3] | 0.827 | 1.59E-006 |
| Pharynx, nos [C14]; ANY morphology | 1.000 | 1.89E-005 |
| Pharynx, nos [C14]; squamous cell carcinoma, nos [8070/3] | 1.000 | 1.89E-005 |
| Skin [C44]; malignant melanoma, nos [8720/3] | 0.189 | 4.27E-006 |
| Skin [C44]; mycosis fungoides [9700/3] | 1.000 | 1.00E-006 |
| Small intestine [C17]; ANY morphology | 0.969 | 5.56E-005 |
| Small intestine [C17]; gastrointestinal stromal tumor, malignant [8936/3] | 0.969 | 5.56E-005 |
| Unspecified digest. organs [C26]; ANY morphology | 1.000 | 0.027 |
| Unspecified digest. organs [C26]; enteropathy type t-cell lymphoma [9717/3] | 1.000 | 0.027 |

*Note*: Data were generated from IntOGen with the corrected *P* value (FDR) of significance indicated. The significant *P* values (< 0.05) are shaded in color.
