# Supplementary material for: Proteomic Analysis Shows Constitutive Secretion of MIF and p53-associated Activity of COX-2−/− Lung Fibroblasts
Source: Genomics Proteomics Bioinformatics. 2017 Dec 13;15(6):339–51. doi: 10.1016/j.gpb.2017.03.005 (PMC5828655; doi:10.1016/j.gpb.2017.03.005)
Supplement: File S1 — GO analysis and enrichment analysis of biological functions. [file mmc8.doc]

**File S1 GO analysis and enrichment analysis of biological functions**

Functional annotation of genes was based on Gene Ontology (GO) (Consortium, 2006; [http://www.geneontology.org](http://www.geneontology.org/)) [55]. Differentially-expressed transcripts (FC ≤ 0.5 or FC ≥ 1.75) generated from [4] (Figures S1−S4) were used for this analysis with each gene listed for its known function. The data were downloaded using Biomart data extraction facility ([www.biomart.org](http://www.biomart.org/)) and KEGG database [57]. The downloaded data were presented into two-column files, containing genes in the first column and the corresponding function in the second column. These files are named “module” files and contain all the genes and their known function from GO database.

The second set of the file was termed as “data” file, which is a binary file. Here the first column represents genes and the second column contains value either “1” or “0”. One (1) indicates that the gene is differentially modulated in the experimental condition(s) in question, whereas zero (0) indicates that the gene is not differentially modulated when compared to WT cells. We listed all the genes that were analyzed in microarray chips (Figures S1−S4; [4]). We used these two files for over-representation or enrichment analysis (EO) of differentially-modulated genes for GO categories including GO Biological Processes and GO Molecular Functions.

EA (overrepresentation) was performed to identify GO categories (Biological Processes or GO Molecular Functions) that might be followed by, *i.e.*, associated by, the upregulated or downregulated genes, *i.e.*, differentially modulated genes, significantly more than expected by chance. In search of statistical significance (*P* value) we use binomial distribution, and *P*-value was calculated as:


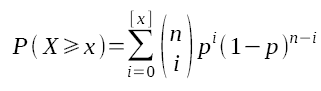


where ***n*** represents a total number of genes in the category, *x* represents the number of differently expressed genes in the category, and *p* represents the frequency of upregulated or downregulated genes.

The resulting *P* values were adjusted for multiple testing using the Benjamin−Hochberg method of false discovery rate (FDR) [60]. We used Gitools ([www.gitools.org](http://www.gitools.org/)) [58] for the entire analysis. Gitools exports heat map, in which column represent the experimental condition(s) in question and rows represent GO terms (modules). Each cell represents the corrected *P* value for the over-representation analysis and presented on a color-coded scale [56,58].
