# Supplementary material for: Proteomic Analysis Shows Constitutive Secretion of MIF and p53-associated Activity of COX-2−/− Lung Fibroblasts
Source: Genomics Proteomics Bioinformatics. 2017 Dec 13;15(6):339–51. doi: 10.1016/j.gpb.2017.03.005 (PMC5828655; doi:10.1016/j.gpb.2017.03.005)
Supplement: File S2 — Extraction and bioinformatic analysis of cancer. [file mmc9.doc]

**File S2 Cancer data extraction and bioinformatics**

IntOGen database was searched to identify different types of cancer, where COX-2 and MIF were significantly upregulated or downregulated [59]. Similarly, these databases were also searched to identify neoplastic conditions where there is a significant gain of MIF and/or loss of COX-2 at the genomics level. We have outlined the types of cancer in a table with their respective *P* values of significance as extracted directly from IntOGen database using Biomart data extraction facility [56,59].

The IntOGen database is a resource to compare and integrate a significant amount of cancer genomic data. IntOGen database harbors and facilitates the detection of the most recurrent alterations that drive tumorigenesis. It collates, annotates, and analyzes high-throughput data about transcriptional, genomic, and mutational changes that have taken place in tumors from different studies annotated with specific cancer types. Currently, it contains 118 studies for mRNA expression profiling and 188 studies for genomic alterations covering 64 different tumor topographies.

Biomart is a portal for IntOGen, providing easy access to various types of data and facilitating the bulk download of all the analysis results [58,59]. The data in IntOGen consist of publicly-available cancer genomic studies collected from databases such as the Gene Expression Omnibus (GEO), ArrayExpress, COSMIC, Progenetix, the Sanger Cancer Genome Project, and the data portal of The Cancer Genome Atlas (TCGA). Each study contains results from high-throughput analyses of numerous human primary tumor samples in comparison with normal cells (normal cells of the same tissue in the case of expression) related to one or more types of cancer for a specific alteration.
